# Anti-stigmatizing: a collaborative autoethnography on recovery from depression

**DOI:** 10.3389/fpsyt.2024.1360967

**Published:** 2024-04-16

**Authors:** Danlei Zhu, Keyi Lyu

**Affiliations:** ^1^ Institute of Vocational & Adult Education, East China Normal University, Shanghai, China; ^2^ Jing Hengyi School of Education, Hangzhou Normal University, Hangzhou, China; ^3^ Chinese Education Modernization Research Institute of Hangzhou Normal University, Hangzhou, China

**Keywords:** depression, recovery, collaborative autoethnography, anti-stigma, qualitative research

## Abstract

**Introduction:**

Despite extensive research on clinical treatments for depression, there remains a significant gap in understanding of the lived experiences and recovery journeys of those with depression. This study sought to explore the recovery process through an “anti-stigmatizing” lens, emphasizing the cultural–psychological mechanisms at play and the importance of personal narratives in shaping the recovery trajectory.

**Methods:**

Using a collaborative autoethnographic approach, this report focuses on the first author’s journey of depression recovery. This research methodology allows for an in-depth exploration of subjective experiences, with a specific emphasis on the interaction between societal stigma, personal identity, and mental-health challenges.

**Results:**

It is found that the depression-recovery experience can be divided into four stages from an anti-stigma perspective: (1) encountering the public stigma of emotions; (2) internalizing the stigma to a self-stigma; (3) “decriminalizing” the expected stigma of a “depressed” identity through diagnosis; and (4) being able to cope with and understanding the public stigma relating to depression when facing it again. Key factors that were found to contribute to recovery were self-awareness, community empowerment, and recognition and acceptance by close friends and family.

**Discussion:**

We propose a reconceptualization of depression that incorporates a societal perspective on internalized stigma. Recovery from depression is not merely a medical process; it also pertains to how the patient frees themselves from public stigma. The results strongly indicate the need for a paradigm shift toward a more inclusive and empathetic approach to mental-health care, and we emphasize the importance of personal narratives in depression recovery.

## 1 Introduction

Depression stands as a formidable challenge in global mental health. According to the World Health Organization, depression affects over 280 million people worldwide (1). Furman and Bender (2) noted that depression “is so prevalent that it has been referred to as the ‘common cold’ of mental illness.” Particularly since the COVID-19 pandemic, the prevalence of depression has been rising, making it a leading cause of disability and death (3).

Much of what is known about depression and recovery from it is based on medical research, and very little is known about the process from the perspective of someone who has experienced depression firsthand. In medical research, depression is often conceptualized as a clinical disorder with a specific set of symptoms that can be identified and treated (4, 5). The dominant approach in contemporary medical science views depression through the lens of a biological model, focusing on physiological factors such as neurochemical imbalances, genetic predispositions, and brain-structure anomalies; common interventions include medication (6, 7), positive-thinking activities (8), and social support (6, 8, 9). However—whether deliberately or otherwise—previous studies have tended to ignore the rich subjective experiences, feelings, and thoughts of people with depression.

The latest research has brought to prominence the importance of incorporating personal narratives into our understanding of depression. For example, the work of Karp (10) and McCue et al. (11) highlights the usefulness of a patient-centric methodology, in which treatments are designed to directly address experiences and needs. In addition to being a necessary component for getting to the core of depression, this outlook is also critical to making treatment methods more humane. This perspective is crucial not only for obtaining a comprehensive understanding of depression but also for developing more empathetic and effective treatments. The research of Lakhan et al. (3) and Salari et al. (12) supports this perspective, showing that such an approach can lead to better outcomes for patients. They emphasize the importance of recognizing and addressing the personal and social dimensions of depression, which often go beyond clinical symptoms. This is particularly important given that, as Villanti et al. (13) notes, relapses and residually impaired social functioning can persist even after clinical recovery has been achieved. These outcomes result in a heavy burden on both the individual concerned and society at large. Additionally, some scholars have proposed new therapeutic approaches for depression based on patients’ personal experiences. For example, Salonen has introduced the Flow with Nature Treatment, which emphasizes the therapeutic potential of engaging with natural environments to enhance mental well-being and alleviate depressive symptoms (14).

However, an essential aspect of the discourse on depression recovery that merits further exploration is the recovery perspective itself. Pioneers like William Anthony, Patricia Deegan, Mike Slade, Mikal Cohen, and Marianne Farkas have advocated for a more holistic understanding of recovery, emphasizing that it is not merely the cessation of symptoms but a deeply personal, ongoing journey of growth, empowerment, and reintegration into society (15–21). Recovery, as defined by these scholars, encompasses not just the alleviation of symptoms but also the restoration of identity, meaning, and purpose in life beyond the confines of mental illness.

From the recovery perspective, the process is inherently individualized, with each person’s journey being unique. (22) describes recovery as a deeply personal process of changing one’s attitudes, values, feelings, goals, skills, and roles to develop a satisfying, hopeful, and contributing life, even within the limitations caused by illness. Patricia Deegan’s work further emphasizes the importance of hope and self-determination in the recovery process, advocating for a shift from being a passive recipient of care to an active agent in one’s healing journey. Recovery-oriented approaches highlight the significance of personal narratives, community support, and empowerment, underscoring the multifaceted nature of healing from depression.

Recovery from depression encompasses a multitude of possible forms, as recent literature suggests (20, 21). Previous descriptions of recovery have often focused on the individual’s perspective, neglecting the interaction between the individual and their social environment and culture. Moreover, the research perspective has been deficient in incorporating diverse cultural viewpoints. In light of this, based on a collaborative autoethnographic discourse between the first and second authors, this paper explores the dynamic process of recovery for an individual with depression. In this study, we attempt to excavate one of these underappreciated individual stories; we draw on the concept of stigma theory to offer an autobiographic depiction that explores how depression is experienced and encountered within society at large. In this way, the authors attempt to discover how the everyday experience of social conventions—combined with individual strength and awareness—is part of a person’s overall recovery. Following our exploration of self-awareness, community, and the daily encounters with stigmatization, we have named this process “anti-stigmatizing.” We aim to contribute to a growing discussion on mental health that stresses the importance of individual stories in raising awareness and revamping society’s thinking about mental illness.

## 2 Literature review on recovery from depression

In the multifaceted landscape of mental-health research and treatment, depression is viewed through various lenses, each contributing distinct perspectives to our understanding and approach to this complex condition.

In the realm of medical science, depression is predominantly conceptualized as a clinical condition with an identifiable set of symptoms, and it is primarily treated through pharmacological and therapeutic interventions. This perspective, which has been largely standardized within the framework of the Diagnostic and Statistical Manual of Mental Disorders (DSM-5) by the American Psychiatric Association, focuses on symptomatology rather than individual lived experiences. The DSM-5 categorizes depression as a distinct mental disorder, thereby orienting treatment toward a more passive role for the patient in the healthcare process (23).

According to previous research, depression manifests in three different and distinct ways: cognitive, social, and motivational symptoms. Cognitive symptoms include a “lack of concentration, pessimism, self-blame, self-dislike, and lack of energy” (24); social symptoms can include difficulties in “making friends and assertiveness”; and motivational symptoms include issues of “dependency and loss of initiative” (25). Recovery in medical research often focuses on the alleviation of these clinical symptoms. Treatment modalities commonly include pharmacotherapy, such as antidepressants (26, 27), and electroconvulsive therapy for severe cases (28). The goal of these treatments is to correct the presumed underlying biological dysfunctions, thereby reducing symptoms and improving a patient’s quality of life (29).

This approach relegates the subjective experiences and societal contexts of individuals to the periphery, prioritizing biological explanations and treatments. Although the physiological basis of depression is now much better understood through the development of drugs and the study of the brain (30, 31), and a large number of medications have been developed to alleviate the symptoms, this has come at the expense of a fuller understanding of the individual and his or her social environment. Psychological approaches to depression tend to delve deeper into the individual’s mind, exploring cognitive patterns, emotions, and behavior. Cognitive behavioral therapy, for instance, treats depression as being a result of negative thought patterns, aiming to modify these patterns to alleviate symptoms (32, 33). However, this approach can sometimes underemphasize the patient’s active role in shaping their recovery journey. In both of these approaches, the depressed person is a symptomatized and labeled as an individual or even object that needs to be controlled.

In contrast to the medical-science and psychological models, the sociological perspective views the individual with depression as a labeled entity within a broader societal context. This lens critiques the medicalization of depression, arguing that the Western medical “gaze” often pathologizes certain mental and physical states, especially those deviating from social norms (34–37). There have been studies specifically examining depression in older adults, new mothers, immigrants, and other identity-based social situations (38–41); most of these studies have focused mainly on the formation of depression and less on the recovery process. This field explores how societal factors—such as socioeconomic status, gender, and race—influence the prevalence and perception of depression. But, as (42) points out, sociological research often analyzes models of depression based on broad patterns in society and does not pay enough attention to autonomous individual agency.

Depression studies use health communication to analyze how society perceives depression. This spans everything from how depression is represented in the media and public-health messaging initiatives to contacts between patients and doctors (43–46). Research into this problem is based on the fact that the ways in which the media characterize depression and other mental disorders affect people’s perceptions of them. This raises the question of whether there is any possibility for depressed individuals to accurately perceive their own self-images (47). The problem with this research method is that the content of mass-media reports about depression and depressed people treats them as indirect objects that are affected by the media without seeing the individuals concerned.

While all these views offer valuable insights, they also tend to build a picture of depression that is divorced from the real-life experiences or choices made by those living with it. In mostly focusing on patients from the perspective of their illness, these methods may even help to reinforce a storyline in which patient activity is largely passive. In view of these circumstances, some recent research has started to shift emphasis toward considering the patient’s voice in explaining and treating depression. For instance, Bury’s (48) *Health and Illness in a Changing Society* argues for making the patient more central to medical decisions. There is often extreme suffering behind illnesses like depression, and this can only be resolved if one understands personal stories of emergency care. Only then can future research and practice relating to depression obtain a more complete understanding of the illness, further respecting and endorsing the patient’s role in treatment.

Hence, we concur with Arthur Kleinman’s model of the depressive experience as a psycho-cultural process (49). The fact that depression is associated with a relationship between the individual and society suggests to Kleinman (50) that it stems from diverse social contexts. Moreover, it is a social emotion as well as an illness (2008). To express how strongly attachment plays into this, the new paradigm for depression research establishes a three-way interactive relationship between body, self, and society, which is strongly shaped by local history. This requires a close look at the building blocks of depression’s recovery experience on a much deeper level.

Our understanding of recovery from depression in this study comes from a definition based on psycho-cultural affirmation, i.e., the resistance to stigma by those with depression trying to live their lives normally. Goffman’s (51) central insights are the foundation of our current conceptions about stigma. Goffman considers stigma a process in which others’ reactions taint a person’s normal identity. The main concept underlying Goffman’s theory of stigma is that people with a “contaminated identity” or “discredited status” will alter how they handle their social intercourse and view themselves (51). This gives a sturdy foundation for thinking about the society in which those with depression operate.

Depression is entangled with social stigma. The depression-care literature is full of stories on the interactional aspects of experiencing this illness. But this struggle is not just against the illness: it also involves an outside world full of prejudice and misunderstanding (52). Research indicates that people with depression and other people with mental illness are considered unpredictable, even dangerous (53). Furthermore, people with depression are usually stigmatized as incurable, weak, and unable to communicate (54). These stereotypes might cause depressed people to perceive themselves as less lovable, more flawed, or inferior (55). They may even feel guilty that they lack the courage to fight off their illness or consider themselves failures for allowing their lives to be limited by it (56). Stigma from society often leads to a lack of awareness, alienation, and even discrimination by others who are aware of someone’s depression (57). The result is that with these social obstacles in place, people living with depression often have a more difficult time recovering (58).

The story of “othering,” which resonates through much existing academic research, further suggests that there is a cleft between the depressed self and social institutions such as schools (59). Works such as *The Social Psychology of Stigma* stretch this split out to the process for recovering addicts (60). Nevertheless, these investigations are more concerned with describing the condition itself or sketching out general social reactions to it rather than delving into individuals’ experiences of stigma.

The experience of recovery from depression is conceived as an anti-stigma process, the aim being to overcome a sense of stigma and reestablish self-identity. The core of this perspective is the relationship between society’s attitudes of prejudice against depression and the individual stories of recovering patients. This also enables us to see people as being actively involved in overcoming society’s stigma. A key idea guiding this study is explaining the journeys that people undertake to break through these barriers and how they regain control over their stories and stand up against depression. In this view, the work focuses on showing how people struggling with depression redefine themselves and resist stereotypes about them, as well as trying to cultivate agency in themselves.

## 3 Materials and methods

### 3.1 A collaborative autoethnography approach

In recent years, there has been a major shift of paradigms in academic research toward real-world experience. In this section, I (the first author) present my journey with depression in the first person through stories and anecdotes in the hope of illuminating the complexities of this ailment and its intricate interplay with societal underpinnings.

The methodological framework for this investigation is autoethnography. Autoethnography (61) describes and systematically examines the personal experiences of a researcher to understand their cultural experience. From disparate elements, this methodological decision weaves together the deeply human story of someone whose life is imbued with issues of wider concern about societal values, prejudices, and problems, and it offers an opportunity for intimate expression. This is helpful for understanding depression within the overall framework of societal views and behavior.

For this purpose, I have taken entries from personal documents, reflections in journals, and expressive letters and statements by close friends (62). These form the narrative’s backbone, offering an uncensored window into my psyche, set against a year that was characterized by global disruptions and intimate tribulations.

While autoethnography relies on an integration of personal reflection and cultural analysis, this does not mean that one can simply tell a story. Rather, autoethnographers must distance themselves from personal experience to reflect upon the ways in which the said experience relates to larger cultural patterns. An autoethnographer thus uses personal experience to make unique and unfamiliar aspects of social life familiar to both insiders (i.e., those with similar experiences) and outsiders (i.e., those without a shared point of reference). One way to accomplish this is through collaboration.

Following Chang (63), collaborative autoethnography is the process of engaging in autoethnography with others at varying levels of participation. In some cases, for example, researchers experiencing the same phenomena will collaboratively create reflections (61, 64), discuss emerging themes, and compose analyses throughout the entirety of the project. Alternatively, one or more researchers may document deeply personal experiences and then solicit other researchers for analysis and interpretation.

This study employed a collaborative autoethnographic model, wherein the first author documented personal experiences and reflections and subsequently engaged in an in-depth interview with the second author. This dialogue was initiated through a series of four comprehensive interviews, each serving to elucidate and expand upon the narratives presented in the autoethnography. The first interview, held on 15 July 2023, lasted for 40 minutes and focused primarily on digging deeper into topics brought up in the autobiographical report; “stigma,” “community,” and “media” were thus explored in this session. These layers provided the interpreters with a deeper understanding of the narrative’s structure. The next interview lasted more than an hour and produced a transcript of 12,000 words. The conversation here centered on a more detailed examination of “stigma” as the interpretive through-line for the material autobiography. A third in-depth thematic interview was conducted on 18 September 2023; the resulting material was 9000 words in total. The second author extended the first part’s study of their battle against stigma. This session brought forth deeper insights about different kinds of stigma and techniques for resisting them at various stages. Our last interview, on 20 October 2023, lasted as long and produced as much material (9000 words). It focused on the characteristics of these stages and how they interacted with social identities. Such a progressive, step-by-step approach deepened our autoethnographic content and gave us an all-embracing structure for discussing and interpreting the multifaceted forces of stigma in everyday life.

### 3.2 Recovery from depression: the fight against stigmatization

In setting out on this autoethnographic journey, I first need to map the lines of my path. I was born into an ordinary family in rural southeast China. My father, who was very gifted in school, had to stop further studies because of a disability. As I grew up, my grandmother used to tell me of her hopes that “if you become a university student, you will have meat to eat every day.” In this kind of family environment, I was the first and only female in the family to ever attend university. Furthermore, I exceeded this dream in 2021 when I was accepted to pursue Ph.D. studies. In addition to representing personal success, this was also a guiding light and a beacon of hope for my community. Nevertheless, joy is often accompanied by shadows. The turbulent March of 2022, which was filled with lockdowns imposed due to the COVID-19 pandemic, the heartbreak felt at the choice between losing a loved one in death or leaving them alive for yet another year, failing grades on lab examinations, and cheating scandals from within interpersonal relationships, left me feeling like I was “bottoming out” emotionally. By May of 2022, this downward spiral had resulted in clinical depression.

Now, driven by boundless optimism and a determination that is all about thriving or failing, I am beginning to see signs of recovery in my mental health. I recall these feelings starting to emerge around December 2022. Beyond being an account of personal struggles, this autoethnography touches on the relationships between perceptions in society and insights into psychiatric patients with mental illness, as well as exploring some reflections about healing.

#### 3.2.1 The notion of abnormal: “I feel myself strange and so negative”

On 15 March 2022, my grad school went into lockdown amid a notable resurgence of COVID-19. Three days were originally planned for maintaining the blockade around the city, but it was to continue with ongoing uncertainty. Inside my six-person dorm, I ate boxed meals over and over again. The news of a desperate city that came through the internet made me feel even sadder and more anxious for civilization. My emotions continued to worsen as the days went by. By 21 March, I had lost most of my appetite and was living on just five grains of rice a day. On 23 March, the physical suffering began—vomiting, diarrhea, and an inability even to bear seeing food. However, at the same time, increasing stories of general famine made me feel guiltier about wasting even a grain.

In a state of physical and emotional turmoil, I confided in people who were close to me, only to hear belittling comments: “You are a national talent. It’s not like being locked up. Why the melodrama? You must be strong!”; these made me feel guiltier and even insincere. Trying to redeem myself, I first looked to friends for help, but they only brought about self-hatred. To try and save them from “contaminating negativity,” I started avoiding contact with other people as much as possible.

On 16 April, the ending of an intimate relationship threw me into a silent storm. I was shaking with desperation and powerless to break free from my confusion. Looking out the window and seeing birds happily flying from tree to tree made me envy them a little. The breakup left me unable to focus or even think clearly. Completing the tasks set by my mentor became an impossible challenge. I felt like someone who had long ago forgotten all their martial arts skills, and I wanted to use this event as an escape from this abnormal body of mine. But this wish was met with opposition from my parents, who did not understand what I had been through emotionally. They believed that leaving school was unnecessary, arguing that the environment outside the school was even more perilous and that facing my school challenges was crucial for my growth.

On 21 April, the students were given a glimmer of hope: the school gave them special permission to leave with parental consent. Anxious for the promise of freedom, I went to see my parents; they did not understand and were afraid. Their words—denying me my sanity, insulting, and belittling my emotional problems—crushed the little glimmer of hope that was there. “Are you crazy? You come back for what? Now you have food and drink in your apartment! Do not be too pretentious.” They could not help heightening the feelings of loneliness and helplessness within me. Once more, that sense of freedom promised by imminent escape from the stifling surface environment present in the school seemed to recede just beyond my grasp. Even worse, I realized that those I considered close to me, none could truly understand the turmoil I was going through.

#### 3.2.2 Unacceptable “incompleteness”: I want to get rid of this illness as soon as possible

The overwhelming pain, guilt, and despair I was experiencing led me to unconsciously inflict harm upon myself. Discovery by a concerned roommate prompted intervention from teachers, parents, and counselors, and on 27 April, I left school, unaware that my journey through illness had just begun. Isolated in a hotel room after my departure, guilt and shame consumed me daily. Existing conditions worsened, and I grappled with early awakenings, heart palpitations, and a persistent desire to self-harm. Each day became a relentless cycle of waking in the early hours, crying, and shaking with fear until evening.

A poignant diary entry from 30 April encapsulates the profound emotional turmoil that gripped me: “It was the 4th day I woke up at 3:30 am. Soon, palpitations and panic enveloped me like a suffocating cloth, leaving me powerless to do anything but shiver and cry. The world remained asleep, and I found myself trapped in an overwhelming panic, unable to control or comprehend myself, incapable of focusing on anything else. I felt a deep sense of disappointment, fear, and confusion about myself, viewing myself through the lens of a monstrous existence.”

After penning these raw emotions, I turned to my phone and, in desperation, began searching on Baidu for answers to my distressing sleep patterns. The search results suggested a connection to depression. As I delved deeper, accidentally clicking on related searches, I was startled to find that every symptom matched my own experiences: daily crying, pain, weakness, self-injury, inability to concentrate, and self-loathing. This realization deepened my fear and self-loathing, casting a shadow on my understanding of my mental state. I recalled the news related to depression that I once saw on social media platforms, “suicide without warning,” “lifelong medication,” “psychiatric hospitals,” and so on. These narratives made me even more scared and desperate, and I blamed myself more seriously, wondering “Did I do something to deserve it?”

I felt like I was being controlled by one of Harry Potter’s Dementors, and I sought the shelter of sharing my pains and fears on a family WeChat group. Nevertheless, a broken-hearted dismissal resulted from their responses after five days. They made a phone call to me, intimating their inability to deal with my “negative energy,” and they asked me to stop sharing. Confused by pain, I asked my friends for help once more. In my time of need, they consistently offered their support through phone calls, some of which occasionally lasted up to half a day. These extended conversations were not only a testament to their genuine concern for my well-being but also a preventive measure against any further self-harm. Their unwavering presence allowed me to escape from the isolation of being unheard and unacknowledged. This strong fellowship became a lifeline, supporting me in my darkest moments, in which I felt hazardously near the edge.

On 2 May 2022, influenced by one of my friends, I attempted counseling in the form of a video call. In this transformative experience, a painful moment left a memorable mark on my heart. With my head lowered, tears fell out and my heartache was released. When I lifted my head again, my counselor was weeping with me. It was then that I realized for the first time that I could show grief and convey my pain freely.

Using an online platform, I completed a self-survey scale, and I found that my results were consistent with depression. Initially, I was considered to be mildly depressed. This diagnosis gave me a sense of safety, “Oh, I’m not a bugbear, I’m just depressed.” Nonetheless, after some days, a more serious categorization was produced by a re-assessment: major depressive disorder. As a result of abruptly receiving this news, I felt horrible, and I was terrified that my condition was doomed to continue to deteriorate. Knowledge of the severity of my symptoms made me sure that improvement was out of reach. In my hopelessness, I spilled my heart to my counselor on the second day with tears in my eyes. When I was weakest, I asked a key question: “Will I recover?” The counselor replied responded firmly, “Yes.” To this day, I regard that event as an essential comfort in which there existed hope in the dark.

The counselor asked me about my emotions and the reasons behind them and said, “Yes, everyone feels helpless and scared in such a situation, but you just need the care that everyone needs. You are not abnormal.” “The scale just means you’re having a hard time right now; it doesn’t represent your condition or who you are.” In that moment, a sudden sense of relaxation washed over me. Major depression was normal, and I was okay—I was normal. By this point, I had already lost 11 kg, a physical testament to the toll of my emotional struggle. I felt like I was teetering on a cliff, and my friends and counselor acted as a branch, pulling me back from the edge.

On 11 May, I returned home, a place where public displays of emotion were discouraged. Despite my parents’ fervent cooking, I lacked the appetite to partake in their hearty dishes. Invitations to go for a walk went unanswered as I grappled with a pervasive sense of weakness. After a week, my father’s frustration erupted, and he demanded to know how long it would take for me to get better and questioning my tears during meals. “I can’t help it, I don’t know how long it will take, I just can’t smile,” I said. His rage transformed into four slaps after my explanation that I could not try to smile. My self-hatred increased and I self-harmed more, and it became abundantly clear that my struggle could not be understood by my parents. This led to me feeling numb, sorrowful, and irritated.

Seeking shelter in friends’ homes across four cities, a journey was started that uncovered the separation between my inner world and the laughter surrounding me. In spite of my capability to mimic suitable emotional reactions, I could not perceive them authentically. This road trip was like a roller coaster: sometimes I felt pulled back from the brink or strove to snap out of it.

In the face of the suffering that I was experiencing at that time, I was determined to go to the hospital. On 24 May, with the support of a friend, I called the psychological health center for the first time. I was advised to repeatedly visit for drug adjustments, which highlighted the significance of periodical follow-up for non-long-term residents. On 25 May, I went with my mother to the city’s hospital psychiatry department, where I had a two-hour consultation and was given a prescription, much to my mother’s evident consternation and rage. The doctor made a definite diagnosis that I suffered from depression and also prescribed paroxetine, advising periodical follow-ups.

The diagnosis gave me a sense of certainty and mild relief. Summoning the courage to start medication, I contemplated contacting my parents and the school to ask to take a break, but my parents had forbidden me from doing so. Feeling indebted to them, I lacked the strength to resist, as if I could not repay their debt of affection. Fortunately, after the diagnosis, my parents ceased their insistence on a rapid recovery and forced laughter.

However, after ten days of medication, intensified side effects such as palpitations led me to stop taking it. My reluctance to seek a medication change stemmed from the emotional and psychological toll of my previous visit to the doctor.

#### 3.2.3 Shifting perceptions: depression is not “me”

After receiving the diagnosis, I returned home, overwhelmed by the sudden surge of emotions I had experienced and fearing their intensity. Spending my days at home, I found myself lacking any motivation to leave my bed. It dawned on me that my current life’s state was eerily similar to being under strict control. Despite having moved beyond that particular period and environment, I felt imprisoned in the past, unable to break free, as if I were trapped in a time capsule. Furthermore, I did not expect anyone to want to be with me anymore, so I shut myself off.

In an attempt to break free from this emotional confinement, I sought refuge at a friend’s house. Living there exposed me to various situations, including an instance during which I lost control and wept in front of my friend. Upon regaining composure and apologizing, my friend expressed confusion about my apology. I explained that I felt I should not have projected negative energy onto them, even though I could not control myself, and I felt sorry and disgusted with myself. In my understanding at the time, my negative energy was unforgivable and would be rejected by everyone. I was prepared for my friends to drift away from me. But to this, my friend responded, “There’s no such thing as should or shouldn’t; you’re fine just the way you are.” I vividly recall the evening sun’s exquisite beauty, perceived by my friend as nature’s loving-kindness. From that day onward, I found solace in silence and was finally able to embrace the present moment. Each day during that period revolved around deep breaths and experiencing the love that nature bestowed upon me. I decided to release all responsibilities and ambitions, allowing time to pass as I lived in a state akin to that of a plant.

Nevertheless, my journey was marked by challenges. Concerned friends bought me a kitten, believing the claims presented on the internet that having a cat or dog could alleviate depression. However, upon returning home, the kitten’s constant purring made me feel disliked and blamed for neglecting her. I also questioned my ability to care for her, feeling as though I was shortchanging the cat. Three days later, devastated, I returned the kitten to a friend.

My state and emotions still go back and forth, and I often need to talk about that time and the emotions I felt then. I started reading *The Power of Self-Care*, *The Amazing Me*, *Seeing Darkness*, and a host of other depression-related books. At first, this was just to pass the time, but in these books, I could feel the empathy and comfort of their authors. It was also in the books that I gradually came to understand what was happening to me, which made me finally stop being so afraid of myself. I realized how cruel I was to myself, how little I knew about my emotions, and how often I “flashed back” to painful situations and needed to recognize these emotions and let myself know that I was being pulled back, not that I was in the situation I was in now. But I just found it frustrating that I still did not have the strength to change. Simultaneously, my appetite improved, and a heightened awareness of food rekindled a sense of desire, reminiscent of being an animal once again.

#### 3.2.4 Living with depression: becoming a more whole “me”

My friends, knowing my love for reading, discovered that there were support communities hosting book clubs specifically for people with depression. They highly recommended and supported my participation. With some skepticism, I joined one of these gatherings. This was a fortuitous turning point during my life journey, and it exposed me to numerous stories similar to my own. This discovery functioned as an efficient antidote for my deep-rooted emotions of shame and isolation, and it cultivated a sense of belonging and shared experience. This did not just entail finding comfort within others’ stories; it also awakened a far-reaching awareness of the pervasiveness of human suffering. It exposed the stereotypes regarding the isolation triggered by depression, uncovering a more extensive narrative in which my experiences were neither alien nor distinctive. In this newly discovered connection with others, a solace was offered, along with a strong verification of my emotions and experiences. This highlighted the significance of community during the healing procedure, which demonstrated how shared experiences were capable of cultivating resilience, as well as a corporate sense of hope. This recognition was key to reforming my feeling of depression from a lonely fight to a shared trip, in which the burden of recovery could be prominently lightened by others’ comprehension and sympathy.

In a key book-sharing activity, I encountered one recovery volunteer whose opinion on psychological illness was far-reaching. Our conversation had a deep resonance, which challenged the conception that psychological illnesses indicated inability or inferiority; on the contrary, they can be regarded as a signal, distracting attention from aspects of our past potentially needing to be considered and rectified. The intrinsic transformative potential to conquer depression was stressed by the volunteer, and the depression was depicted as a journey to a more complete self rather than the return to a former state. This information had a far-reaching resonance, and it enabled me to view myself as not “abnormal” or monstrous, but instead as somebody on the road to recovery and self-discovery.

Going back to the academic arena after a rest, I found that my early departure had made the school community take note of my poor psychological health. Because I did not hide my identity as a depression patient, those around me still looked at me as if I were a monster; I became the closest person to an otherworldly being among them. Whenever there was any news about a depression patient, such as a suicide, they would send me messages, inquiring about my situation. At the same time, others would compare my behavior to the symptoms of depression, and whenever I seemed even slightly happy, I would receive comments like “You don’t look like someone with depression.” Suddenly, I was reminded of the familiar notion that a person with depression must always be in a state of sadness and pain, unable to experience joy, just as I, as a top student, was expected to always remain calm and joyful, never allowed a moment of pain or breakdown.

Participating in written exchanges and frank discussions with my counselor propelled an important examination regarding my internalized narrations and social expectations. I questioned my strict role and identity, challenging the conception aligning with the utopian image of a “perfect Ph.D.” Feeling extremely vulnerable, I nonetheless accepted the feeling of no shame in my vulnerability. My right to self-estimate was acknowledged, and I did not feel forced to accept everything others regarded as “me” any longer. These internal words and reflections initiated a journey of self-liberation, and this took precedence over my physical health and my accepted imperfections, and I abandoned the quest for perfection. Accepting both my flaws and those of others marked a significant stride in my ongoing journey of recovery and self-acceptance.

## 4 Results

In this section, the narrative perspective shifts to the third-person present tense, using examples from my (the first author’s) experiences to elaborate on the potential stigmatization that a Ph.D. student might face as an individual dealing with depression, along with the potential coping mechanisms they may employ.

Initially, the author adheres to the obedience and acceptance she has always exhibited as a “rule-follower” (65) since childhood and adeptly plays the role of a “Ph.D. student.” During this period, the author’s inner symbolic order aligns harmoniously with social and moral norms. In Chinese social–moral norms, a “Ph.D. student” is perceived as a person of culture and morality—nearly perfect. However, the author fails to recognize that this Ph.D. student is merely a role and not her true self, mistakenly equating herself with the ideal Ph.D. student. Following the shock of the epidemic blockade, the author’s inner symbolic order divides into self-feelings and social–moral norms, causing her to keenly experience the pain of being judged by these internalized norms. Confused and overwhelmed by this self-inflicted pain, the author seeks help from her close relatives, yearning for recognition and approval. Unfortunately, these people fail to comprehend the author’s suffering, believing that individuals with material abundance have no right to experience such hardships. Even the emotion of “pain” is stigmatized within the social norms. As described by Hochschild (66), society establishes norms dictating which emotions are appropriate or inappropriate in specific contexts, guiding how individuals experience and express their emotions.

Consequently, the first author transforms into what Goffman refers to as a “deviant” after encountering stigmatizing comments, such as being accused of “acting” when expressing painful emotions. At this stage, the author faces public stigmatization of her negative emotions. Goffman (51) defines public stigma as the public’s reaction to individuals with certain characteristics or identities that are perceived as socially unpopular or inferior, leading to the author’s painful lack of acceptance by others.

Due to her trust in her relatives, the author internalizes the stigmatizing labels they impose. Unable to confront her vulnerable self, which deviates from societal moral norms, the more the author identifies, the more she fears being labeled a “deviant,” experiencing increased shame and pain. This emotional distress manifests physically, resulting in symptoms including loss of appetite and self-injurious behavior. At this stage, the author fully internalizes the stigma, transforming it into self-stigma and engaging in intense self-punishment. Internalized stigma, or self-stigma, occurs when individuals assimilate and internalize negative societal beliefs and attitudes directed toward them due to association with a stigmatized condition or identity (67). This leads to intense feelings of shame, diminished self-esteem, and a sense of unworthiness. The description of the author’s experiences in the previous section clearly illustrates this phase, showing her overwhelming shame, low self-esteem, and self-perception as a “sinner.” During this period, the author’s primary anti-stigma strategies involve emotional expression and seeking support. Despite emotions being a source of stigma, they serve as a powerful means for the author to shield herself from stigma. Consequently, as the external stigmatization intensifies, the author’s pain and suffering also increase. Confiding in close friends provides her with perspectives that contradict stigmatizing views, offering some relief.

Seeking medical attention for a physically ill body aligns with the author’s internalized societal moral norms. Consequently, she begins to seek medical help, and medical science provides an objective name for the condition: “depression.” While this seemingly objective name temporarily shields the author from the morass of stigmatization, the illness itself becomes a stigma, and the “depressed person” remains a kind of “deviant.” The author begins to feel the stigma of anticipation, which involves expecting future stigmatization, leading to anxiety, fear, and a tendency to conceal one’s condition. This is evident in her reluctance to open up due to fear of misunderstanding and stigma from others (51).

Fortunately, this time, the “deviant” has a clear name, and the author has changed the description from “Harry Potter’s Dementor” to the “Black Dog.” As Hochschild (66) says, naming is a way of shaping reality and exercising power. During this stage, the author’s primary anti-stigma strategies shift toward cognitive adjustment and self-liberation. Unlike the previous stage, the author’s self-perception transforms from an internalized demand to be the “perfect doctoral student” to accepting herself as a “depression patient.” The author begins to embrace her identity, viewing herself as “just an ordinary girl seeking validation,” and she starts learning to be mindful, naming her emotions, and ceasing to seek external rescue and understanding. Additionally, the author’s engagement in community activities opens up possibilities of empowerment, setting her on a path of self-liberation.

There was a prominent change in the standards applied to self-evaluation, which caused the first author’s recovery. Far away from the ethical standards imposed by her family, the author set about assessing her value on the basis of individual emotional experiences. This change indicates a transition from a need for exterior validation to an introspective comprehension of self-value. Additionally, it demonstrates the author’s attempt to reclaim the authority that she has been denied according to the deeply rooted social–ethical system. The author chose to build her self-worth on individual emotional norms, and she efficiently broke through the hegemonic narrative that was controlling her sense of self. This reclamation of assessment ability indicates resistance against the systematic stresses and social expectations that previously formed her identity. It highlights the author’s journey from being subject to societal norms to being an autonomous individual defining her own worth.

Goffman (51) suggests that individuals manage stigma by controlling information about their stigmatized identity, challenging stigma, social-cue navigation, and role-playing. This case argues in favor of the explanatory power of Goffman’s stigma theory. However, unlike Goffman’s stated strategy of managing stigma, the author does not hide her stigmatized identity throughout the process but rather exposes herself to various “authorities” in search of self-definition and recognition. At the root, Bourdieu (68) saw the need for recognition by others as foundational for the human condition and argued that the social world provides “what is rarest, recognition, consideration, in other words, quite simply, reasons for being.” The closer the author feels to the authority, the more she identifies with its definition, regardless of whether this definition is in her favor or not, much like how the counselor’s acknowledgment did not separate the author from stigmatized identification right from the start.

## 5 Discussion

In this collaborative autoethnographic examination, we have delved into the intricate tapestry of a doctoral student’s experiences navigating through the tumultuous terrains of depression and subsequent recovery. The process can be divided into four parts: suffering from the public stigma, internalizing the stigma, “decriminalizing” the stigma through diagnosis, and coping with and understanding the public stigma of depression when encountering it again. This collaborative autoethnographic examination delves into the intricate recovery journey from depression, showcasing not just the struggle with the illness itself but also the profound process of self-discovery, empowerment, and societal reintegration. Aligning with the recovery perspectives highlighted by pioneers in the field, our discussion emphasizes the multifaceted nature of recovery, drawing particularly on Patricia Deegan’s insights into the personal experience of recovery. Patricia Deegan’s first-person deliberations on recovery shed light on the importance of hope, agency, and the active role of the individual in navigating their path to wellness. Deegan describes recovery not as a linear return to a pre-illness state but as a deeply personal journey of developing new meaning and purpose in life, despite the limitations imposed by mental illness (17). This view aligns with the first author’s experiences, as documented in our study, where recovery transcended the mere alleviation of depressive symptoms to encompass a broader quest for self-definition, acceptance, and integration into a community.

Previous research on recovery from depression has predominantly focused on intervention mechanisms such as medications and behavioral therapies (69–75), often overlooking the subjective experience of recovery and how individuals perceive, select, and accept treatments during the process. The creative notion of “anti-stigmatizing” is introduced in this paper to deepen the awareness of depression recovery. Anti-stigmatizing emphasizes the demand for recovery to be regarded as a multi-faceted journey encompassing individual, societal, and cultural aspects beyond mere clinical results. It involves the resistance to stigma by sufferers with depression as they strive to live their lives normally, overcoming societal attitudes of prejudice towards depression and reestablishing self-identity. The process of anti-stigmatizing is conceptualized as an active involvement in overcoming society’s stigma, redefining selves, resisting stereotypes, and cultivating agency. It aims to foster a nuanced understanding of depression that includes personal narratives and the sociocultural dimensions of mental health, working towards creating a supportive, destigmatizing environment for those grappling with depression.

Aligning with the recovery perspectives highlighted by pioneers in the field, our discussion emphasizes the multifaceted nature of recovery, drawing particularly on Patricia Deegan’s insights into the personal experience of recovery. Patricia Deegan’s first-person deliberations on recovery shed light on the importance of hope, agency, and the active role of the individual in navigating their path to wellness. Deegan describes recovery not as a linear return to a pre-illness state but as a deeply personal journey of developing new meaning and purpose in life, despite the limitations imposed by mental illness. This view aligns with the first author’s experiences, as documented in our study, where recovery transcended the mere alleviation of depressive symptoms to encompass a broader quest for self-definition, acceptance, and integration into a community. The first author’s journey underscores the significance of embracing one’s vulnerabilities and transforming them into strengths. This mirrors Deegan’s advocacy for a narrative of recovery that is centered on the empowerment of the individual, moving beyond the patient role to reclaim control over one’s life and identity. The first author’s active engagement in self-reflection, community participation, and the deliberate choice to expose and confront the stigmatized aspects of their identity illustrate the enactment of recovery as an ongoing, dynamic process.

The principal methodology employed in this study was autoethnography, which extends profound insights into the experiential realm of depression. This methodological approach facilitates a veracious depiction of the first author’s journey, surpassing the limitations inherent in traditional research paradigms. The strength of autoethnography lies in its capacity to elucidate the nuanced emotional and mental landscapes, crafting a rich mosaic of personal narratives that unveil the complexities involved in navigating depression. Engaging in autoethnographic writing and reflection allowed the first author to retrospectively examine her experiences from a detached perspective, effectively extricating herself from the immediacy of past emotions. This process not only reinstated a sense of empowerment but also restored her narrative agency, contributing positively to her recovery pathway. Therefore, we wish to highlight the significance and advantages of utilizing autoethnography in the study of mental illnesses or mood disorders, advocating for its wider application within the field to leverage its potential in elucidating personal and psychological complexities.

By venturing into the personal experiences of an individual, this research enables identification of the important factors relating to the formation of societal support systems for patients who are recovering from depression. This investigation underlines the importance of identifying and prioritizing the distinctive demands and individual preferences of depression patients; this is in contrast to previous research with a focus on objective representation (76). The importance of trust and belief within interpersonal interactions between patients and their healthcare givers or support networks has been highlighted. The research results support follow-up work and intervention approaches that give priority to patient-focused methods. Additionally, they indicate that the individual’s voice should be acknowledged in their recovery, and sufficient emotional identification and affirmation should be guaranteed for depression patients.

In cultural backgrounds such as those found in much of China, family and community connections have a prominent influence. This research highlights that efficient and comprehensive family-oriented support systems can boost the recovery of people with depression. An important perspective has also arisen from this work, one that contradicts the idea that families are unwitting catalysts to public stigma or interventions for the recovery of patients with depression. As a result, the fundamental understanding of depression should be expanded beyond those who are immediately affected, and ways to change the perception of traumas and emotions must be deeply researched. This includes cultivating acceptance and acknowledgment of one’s own feelings and those of family members. Psychological-health literacy needs to be improved among members of the public, including the families of those with depression; this will play an indispensable role in changing people’s attitudes to depression. This literacy should include identifying signs of depression, understanding how and when to look for help, and being aware of the diversity of available therapies.

By drawing upon the emerging concepts of recovery, particularly Deegan’s insights into the personal recovery experience, our discussion enriches the understanding of the first author’s recovery process. It underscores the transformative potential of embracing one’s recovery journey as an opportunity for growth, empowerment, and the reclamation of one’s narrative and place within the community. In alignment with this, it is noted that empowerment originates in active community engagement, going beyond mere oral articulation, and it is robustly manifested through taking inspirational and uplifting steps. There is a chain reaction of empowerment that can spread widely, impacting psychological health education in academic institutions and affecting the wider approach to education, particularly in the present post-pandemic age. We thus advocate for a paradigmatic shift, supporting a transition from the age of imposition and demands to another age that fosters inspiration and positive prospects.

Furthermore, we posit that depression can function as a crucible for problem resolution and personal growth; this standpoint focuses on the key function of self-directed study examining how to dismantle the stigma around depression. This standpoint is highlighted by the clear-cut comparison with media depictions of patients living with depression that show them as helpless or prone to self-harm. However, the interior resilience and motivation that can be present in people with depression—even though they may be hidden in the depths of their hearts—have been clarified by this research. We suggest the need to foster a tendency for learning, as this appears to be a robust tactic, while concurrently struggling with stigma and working as a preventative and therapeutic agent for a series of psychological wellbeing challenges. It has been identified that education regarding “giving up” and “failure” is lacking against the background of Chinese culture. This research thus highlights that more inclusive content is needed to address these cultural nuances.

In integrating these concepts of recovery into our discussion, we advocate for a broader understanding of depression recovery, one that recognizes the complexity of the individual’s experience and the essential role of personal agency, community support, and the redefinition of identity. Our study contributes to the discourse on mental health recovery by highlighting the necessity of an empathetic, inclusive approach that values the unique paths individuals take towards healing and wellness.

To sum up, it is clear that in addition to fighting the illness itself, people struggling with depression have to bear the weight of stigmatization. This research demonstrates the possibility of a positive world in which recovering from depression is like being liberated from arduous challenges, and this supports the need for a setting that contributes to growth and healing with no struggle against stigma. By championing a shift in societal attitudes to mental health, our goal is to contribute to the establishment of a more compassionate and effective framework for supporting individuals on their paths to recovery from depression. This involves fostering a nuanced understanding of the patient’s perspective, gaining deeper insights into the sociocultural dimensions of mental health, and actively working toward creating a supportive, destigmatizing environment for those grappling with depression.

## Data availability statement

The original contributions presented in the study are included in the article/supplementary material. Further inquiries can be directed to the corresponding author.

## Ethics statement

Ethical review and approval was not required for the study of human participants in accordance with the local legislation and institutional requirements. This research was conducted as a self-ethnographic study, focusing on the first author’s personal experiences with depression recovery. Given the nature of self-ethnography, where the researcher is also the subject of the research, the study did not involve external participants. Therefore, Institutional Review Board approval was not applicable for this specific study. The research methodologies were designed to ensure confidentiality, respect for the author’s privacy, and adherence to ethical considerations for self-reporting research. All reflections, analyses, and conclusions drawn from the research are based on the first author’s personal experiences, documented with the utmost care to maintain ethical integrity in self-research.

## Author contributions

DZ: Data curation, Formal analysis, Investigation, Methodology, Project administration, Resources, Validation, Writing – original draft, Writing – review & editing. KL: Conceptualization, Formal analysis, Funding acquisition, Methodology, Project administration, Supervision, Writing – review & editing.

## References

[1] World Health Organization. Depression And Other Common Mental Disorders: Global Health Estimates. Geneva, Switzerland: World Health Organization (2017).

[2] Furman R Bender K. The social problem of depression: A multi-theoretical analysis. J Soc Soc Welfare. (2003) 30:123. doi: 10.15453/0191-5096.2920

[3] Lakhan R Agrawal A Sharma M. Prevalence of depression, anxiety, and stress during covid-19 pandemic. J Neurosci Rural Pract. (2020) 11:519–25. doi: 10.1055/s-0040-1716442 PMC7595780 33144785

[4] Beirão D Monte H Amaral M Longras A Matos C Villas-Boas F. Depression in adolescence: A review. Middle East Curr Psychiatry. (2020) 27:1–9. doi: 10.1186/s43045-020-00050-z

[5] Nobis A Zalewski D Waszkiewicz N. Peripheral markers of depression. J Clin Med. (2020) 9:3793. doi: 10.3390/jcm9123793 33255237 PMC7760788

[6] Goldman LS Nielsen NH Champion HC Council On Scientific Affairs, A. M. A. Awareness, diagnosis, and treatment of depression. J Gen Internal Med. (1999) 14:569–80. doi: 10.1046/j.1525-1497.1999.03478.x PMC1496741 10491249

[7] Smith GT Atkinson EA Davis HA Riley EN Oltmanns JR. The general factor of psychopathology. Annu Rev Clin Psychol. (2020) 16:75–98. doi: 10.1146/annurev-clinpsy-071119-115848 32040926

[8] Gelenberg AJ. A review of the current guidelines for depression treatment. J Clin Psychiatry. (2010) 71:26478. doi: 10.4088/JCP.9078tx1c 20667285

[9] Beck AT Alford BA. Depression: Causes And Treatment. Philadelphia, Pennsylvania, USA: University Of Pennsylvania Press (2009).

[10] Karp DA. Speaking Of Sadness: Depression, Disconnection, And The Meanings Of Illness. Oxford, England: Oxford University Press (2017).

[11] Mccue M Sarkey S Eramo A François C Parikh SV. Using the goal attainment scale adapted for depression to better understand treatment outcomes in patients with major depressive disorder switching to vortioxetine: A phase 4, single-arm, open-label, multicenter study. BMC Psychiatry. (2021) 21:1–10. doi: 10.1186/s12888-021-03608-1 34895181 PMC8665619

[12] Salari N Hosseinian-Far A Jalali R Vaisi-Raygani A Rasoulpoor S Mohammadi M. Prevalence of stress, anxiety, depression among the general population during the covid-19 pandemic: A systematic review and meta-analysis. Globalization Health. (2020) 16:1–11. doi: 10.1186/s12992-020-00589-w 32631403 PMC7338126

[13] Villanti AC Lepine SE Peasley-Miklus C West JC Roemhildt M Williams R. Covid-related distress, mental health, and substance use in adolescents and young adults. Child Adolesc Ment Health. (2022) 27:138–45. doi: 10.1111/camh.12550 PMC9018497 35253363

[14] Salonen K Hyvönen K Paakkolanvaara J-V Korpela K. Flow with nature treatment for depression: participants’ Experiences. Front Psychol. (2022) 12:768372. doi: 10.3389/fpsyg.2021.768372 35069344 PMC8766993

[15] Deegan PE. Recovery: the lived experience of rehabilitation. Psychosocial Rehabil J. (1988) 11:11. doi: 10.1037/h0099565

[16] Deegan PE. Recovering Our Sense Of Value After Being Labeled: Mentally Ill. NJ: Slack Incorporated Thorofare (1993). doi: 10.3928/0279-3695-19930401-06 8487230

[17] Deegan P. Recovery as A journey of the heart. Psychiatr Rehabil J. (1996) 19:91. doi: 10.1037/h0101301

[18] Farkas M Gagne C Anthony W Chamberlin J. Implementing recovery oriented evidence based programs: identifying the critical dimensions. Community Ment Health J. (2005) 41:141–58. doi: 10.1007/s10597-005-2649-6 15974495

[19] Leendertse J Wierdsma A Van Den Berg D Ruissen A Slade M Castelein S. Personal recovery in people with A psychotic disorder: A systematic review and meta-analysis of associated factors. Front Psychiatry. (2021) 12:622628. doi: 10.3389/fpsyt.2021.622628 33708145 PMC7940758

[20] Kotera Y Llewellyn-Beardsley J Charles A Slade M. Common humanity as an under-acknowledged mechanism for mental health peer support. Int J Ment Health Addict. (2022), 1–7. doi: 10.1007/s11469-022-00916-9

[21] Kotera Y Rennick-Egglestone S Ng F Llewellyn-Beardsley J Ali Y Newby C. Assessing diversity and inclusivity is the next frontier in mental health recovery narrative research and practice. JMIR Ment Health. (2023) 10:E44601. doi: 10.2196/44601 37067882 PMC10152384

[22] Anthony WA. Recovery from mental illness: The guiding vision of the mental health service system in the 1990s. Psychiatr Rehabil J. (1993) 16(4):11–23. doi: 10.1037/h0095655

[23] American Psychiatric Association D Association, A. P. Diagnostic And Statistical Manual Of Mental Disorders: Dsm-5. Washington, DC: American Psychiatric Association. (2013). doi: 10.1176/appi.books.9780890425596

[24] Wynaden D Wichmann H Murray S. A synopsis of the mental health concerns of university students: results of A text-based online survey from one Australian university. Higher Educ Res Dev. (2013) 32:846–60. doi: 10.1080/07294360.2013.777032

[25] Khawaja NG Bryden KJ. The development and psychometric investigation of the university student depression inventory. J Affect Disord. (2006) 96:21–9. doi: 10.1016/j.jad.2006.05.007 16784780

[26] Kent JM. Snaris, nassas, and naris: new agents for the treatment of depression. Lancet. (2000) 355:911–8. doi: 10.1016/S0140-6736(99)11381-3 10752718

[27] Holtzheimer Iii PE Nemeroff CB. Advances in the treatment of depression. Neurorx. (2006) 3:42–56. doi: 10.1016/j.nurx.2005.12.007 16490412 PMC3593359

[28] Lisanby SH. Electroconvulsive therapy for depression. New Engl J Med. (2007) 357:1939–45. doi: 10.1056/NEJMct075234 17989386

[29] Cohen ZD Derubeis RJ. Treatment selection in depression. Annu Rev Clin Psychol. (2018) 14:209–36. doi: 10.1146/annurev-clinpsy-050817-084746 29494258

[30] Niedzwiedz CL Knifton L Robb KA Katikireddi SV Smith DJ. Depression and anxiety among people living with and beyond cancer: A growing clinical and research priority. BMC Cancer. (2019) 19:1–8. doi: 10.1186/s12885-019-6181-4 31604468 PMC6788022

[31] Maj M Stein DJ Parker G Zimmerman M Fava GA De Hert M. The clinical characterization of the adult patient with depression aimed at personalization of management. World Psychiatry. (2020) 19:269–93. doi: 10.1002/wps.20771 PMC7491646 32931110

[32] Beck AT. Cognitive Therapy Of Depression. New York, New York, USA: Guilford Press (1979).

[33] Huibers MJ Lorenzo-Luaces L Cuijpers P Kazantzis N. On the road to personalized psychotherapy: A research agenda based on cognitive behavior therapy for depression. Front Psychiatry. (2021) 11:607508. doi: 10.3389/fpsyt.2020.607508 33488428 PMC7819891

[34] Foucault M Cooper D. Madness and civilization. (R. Howard, Trans.) (1961). doi: 10.4324/9780203278796

[35] Abrams LS Curran L. Not just A middle-class affliction: crafting A social work research agenda on postpartum depression. Health Soc Work. (2007) 32:289–96. doi: 10.1093/hsw/32.4.289 18038730

[36] Maj M. When does depression become A mental disorder? Br J Psychiatry. (2011) 199:85–6. doi: 10.1192/bjp.bp.110.089094 21804141

[37] Walton QL. Living in between: A grounded theory study of depression among middle-class black women. J Black Psychol. (2022) 48:139–72. doi: 10.1177/00957984211036541

[38] Bai Z Xu Z Xu X Qin X Hu W Hu Z. Association between social capital and depression among older people: evidence from Anhui Province, China. BMC Public Health. (2020) 20:1–11. doi: 10.1186/s12889-020-09657-7 33066764 PMC7565750

[39] Elliott GK Millard C Sabroe I. The utilization of cultural movements to overcome stigma in narrative of postnatal depression. Front Psychiatry. (2020) 11:532600. doi: 10.3389/fpsyt.2020.532600 33192649 PMC7661847

[40] Sidani JE Shensa A Escobar-Viera CG Primack BA. Associations between comparison on social media and depressive symptoms: A study of young parents. J Child Family Stud. (2020) 29:3357–68. doi: 10.1007/s10826-020-01805-2

[41] Jang H Tang F. Loneliness, age at immigration, family relationships, and depression among older immigrants: A moderated relationship. J Soc Pers Relat. (2022) 39:1602–22. doi: 10.1177/02654075211061279 PMC9216219 35747127

[42] Horwitz AV. Creating Mental Illness. (2001). doi: 10.7208/chicago/9780226765891.001.0001

[43] Tao L Jacobs L. “Inbox me, please”: analysing comments on anonymous facebook posts about depression and suicide. J Psychol Afr. (2019) 29:491–8. doi: 10.1080/14330237.2019.1665903

[44] Naslund JA Bondre A Torous J Aschbrenner KA. Social media and mental health: benefits, risks, and opportunities for research and practice. J Technol Behav Sci. (2020) 5:245–57. doi: 10.1007/s41347-020-00134-x PMC7785056 33415185

[45] Mccosker A Gerrard Y. Hashtagging depression on instagram: towards A more inclusive mental health research methodology. New Media Soc. (2021) 23:1899–919. doi: 10.1177/1461444820921349

[46] Hummel AD Ronen K Bhat A Wandika B Choo EM Osborn L. Perinatal depression and its impact on infant outcomes and maternal-nurse sms communication in A cohort of Kenyan women. BMC Pregnancy Childbirth. (2022) 22:1–16. doi: 10.1186/s12884-022-05039-6 36138357 PMC9494796

[47] Wahl OF. Media Madness: Public Images Of Mental Illness. New Brunswick, New Jersey, USA: Rutgers University Press (1995).

[48] Bury M. Health And Illness In A Changing Society. Abingdon, Oxfordshire, England: Routledge (2013). doi: 10.4324/9781315004020

[49] Lee DTS Kleinman J Kleinman A. Rethinking depression: An ethnographic study of the experiences of depression among Chinese. Harvard Rev Psychiatry (2007)15(1):1–8 10.1080/10673220601183915 17364968

[50] Kleinman A. The Illness Narratives: Suffering, Healing, And The Human Condition. New York, New York, USA: Basic Books (2020).

[51] Goffman E. Stigma: Notes On The Management Of Spoiled Identity. New York, New York, USA: Simon And Schuster (2009).

[52] Corrigan PW Watson AC. The paradox of self-stigma and mental illness. Clin Psychol: Sci Pract. (2002) 9:35. doi: 10.1093/clipsy/9.1.35

[53] Schomerus G Schindler S Sander C Baumann E Angermeyer MC. Changes in mental illness stigma over 30 years–improvement, persistence, or deterioration? Eur Psychiatry. (2022) 65:E78. doi: 10.1192/j.eurpsy.2022.2337 36328960 PMC9724218

[54] Woon LS-C Im Khoo S Baharudin A Midin M. Association between insight and internalized stigma and other clinical factors among patients with depression: A cross-sectional study. Indian J Psychiatry. (2020) 62:186. doi: 10.4103/psychiatry.IndianJPsychiatry_612_19 32382179 PMC7197844

[55] Mcginn LK. Cognitive behavioral therapy of depression: theory, treatment, and empirical status. Am J Psychother. (2000) 54:257–62. doi: 10.1176/appi.psychotherapy.2000.54.2.257 10928248

[56] Morgan AJ Wright J Reavley NJ. Review of Australian initiatives to reduce stigma towards people with complex mental illness: what exists and what works? Int J Ment Health Syst. (2021) 15:1–51. doi: 10.1186/s13033-020-00423-1 33461567 PMC7814561

[57] Link BG Phelan JC. Conceptualizing stigma. Annu Rev Sociol. (2001) 27:363–85. doi: 10.1146/annurev.soc.27.1.363

[58] Corrigan PW Wassel A. Understanding and influencing the stigma of mental illness. J Psychosocial Nurs Ment Health Serv. (2008) 46:42–8. doi: 10.3928/02793695-20080101-04 18251351

[59] Luoma JB Nobles RH Drake CE Hayes SC O’hair A Fletcher L. Self-stigma in substance abuse: development of A new measure. J Psychopathol Behav Assess. (2013) 35:223–34. doi: 10.1007/s10862-012-9323-4 PMC3680138 23772099

[60] Major B O’brien LT. The social psychology of stigma. Annu Rev Psychol. (2005) 56:393–421. doi: 10.1146/annurev.psych.56.091103.070137 15709941

[61] Ellis C Bochner A. Autoethnography, personal narrative, reflexivity: researcher as subject. Handbook of qualitative research (2nd Edition) Denzin NK Lincoln YS eds. Sage Publications. (2000), 733–68. Available at: https://digitalcommons.usf.edu/spe_facpub/91.

[62] Sarantakos S. Social Research. London, England: Bloomsbury Publishing (2017).

[63] Chang H. Autoethnography As Method. Abingdon, Oxfordshire, England: Routledge (2016). doi: 10.4324/9781315433370

[64] Ellis C Rawicki J. Collaborative witnessing of survival during the holocaust: an exemplar of relational autoethnography. Qual Inq. (2013) 19:366–80. doi: 10.1177/1077800413479562

[65] Willis P. Learning To Labour: How Working Class Kids Get Working Class Jobs. Abingdon, Oxfordshire, England: Routledge (2017).

[66] Hochschild AR. Emotion work, feeling rules, and social structure. Am J Sociol. (1979) 85:551–75. doi: 10.1086/227049

[67] Nan D Chong ES Dannuo W Zewei L Zexuan M Shuyu D. Prevalence, risk, and protective factors of self-stigma for people living with depression: A systematic review and meta-analysis. J Affect Disord. (2023) 332:327–40. doi: 10.1016/j.jad.2023.01.020 37060952

[68] Bourdieu P. Cultural reproduction and social reproduction. In: Knowledge, Education, And Cultural Change. Abingdon, Oxfordshire, England: Routledge (2018).

[69] De Zwart PL Jeronimus BF De Jonge P. Empirical evidence for definitions of episode, remission, recovery, relapse and recurrence in depression: A systematic review. Epidemiol Psychiatr Sci. (2019) 28:544–62. doi: 10.1017/S2045796018000227 PMC7032752 29769159

[70] Blake J Leppma M Mamboleo G Nauser J O’toole H Chan F. Attachment and hope as A framework for improving depression outcomes for trauma patients. J Appl Rehabil Couns. (2020) 51:94–114. doi: 10.1891/JARC-D-19-00005

[71] Brush CJ Foti D Bocchine AJ Muniz KM Gooden MJ Spaeth AM. Aerobic exercise enhances positive emotional reactivity in individuals with depressive symptoms: evidence from neural responses to reward and emotional content. Ment Health Phys Activity. (2020) 19:100339. doi: 10.1016/j.mhpa.2020.100339

[72] Cuijpers P Stringaris A Wolpert M. Treatment outcomes for depression: challenges and opportunities. Lancet Psychiatry. (2020) 7:925–7. doi: 10.1016/S2215-0366(20)30036-5 32078823

[73] Schuling R Huijbers MJ Van Ravesteijn H Donders R Cillessen L Kuyken W. Recovery from recurrent depression: randomized controlled trial of the efficacy of mindfulness-based compassionate living compared with treatment-as-usual on depressive symptoms and its consolidation at longer term follow-up. J Affect Disord. (2020) 273:265–73. doi: 10.1016/j.jad.2020.03.182 32421612

[74] Büttner CM Rudert SC Greifeneder R. Depressed and excluded: do depressive symptoms moderate recovery from ostracism? J Affect Disord. (2021) 294:730–6. doi: 10.1016/j.jad.2021.07.075 34348168

[75] Strege MV Richey JA Siegle GJ. What does “Staying well” After depression mean? Chronic low grade symptomatology after treatment for depression is common. J Affect Disord. (2022) 317:228–35. doi: 10.1016/j.jad.2022.08.075 PMC10012845 36029878

[76] Lorkiewicz P Waszkiewicz N. Biomarkers of post-covid depression. J Clin Med. (2021) 10:4142. doi: 10.3390/jcm10184142 34575258 PMC8470902

